# A Significant Statistical Advancement on the Predictive Values of ERCC1 Polymorphisms for Clinical Outcomes of Platinum-Based Chemotherapy in Non-Small Cell Lung Cancer: An Updated Meta-Analysis

**DOI:** 10.1155/2016/7643981

**Published:** 2016-01-21

**Authors:** Yali Han, Jie Liu, Meili Sun, Zongpu Zhang, Chuanyong Liu, Yuping Sun

**Affiliations:** ^1^Department of Oncology, Jinan Central Hospital, Shandong University, Jinan, Shandong 250013, China; ^2^School of Medicine, Shandong University, Jinan, Shandong 250012, China

## Abstract

*Background*. There is no definitive conclusion so far on the predictive values of ERCC1 polymorphisms for clinical outcomes of platinum-based chemotherapy in non-small cell lung cancer (NSCLC). We updated this meta-analysis with an expectation to obtain some statistical advancement on this issue.* Methods*. Relevant studies were identified by searching MEDLINE, EMBASE databases from inception to April 2015. Primary outcomes included objective response rate (ORR), progression-free survival (PFS), and overall survival (OS). All analyses were performed using the Review Manager version 5.3 and the Stata version 12.0.* Results*. A total of 33 studies including 5373 patients were identified. ERCC1 C118T and C8092A could predict both ORR and OS for platinum-based chemotherapy in Asian NSCLC patients (CT + TT versus CC, ORR: OR = 0.80, 95% CI = 0.67–0.94; OS: HR = 1.24, 95% CI = 1.01–1.53) (CA + AA versus CC, ORR: OR = 0.76, 95% CI = 0.60–0.96; OS: HR = 1.37, 95% CI = 1.06–1.75).* Conclusions*. Current evidence strongly indicated the prospect of ERCC1 C118T and C8092A as predictive biomarkers for platinum-based chemotherapy in Asian NSCLC patients. However, the results should be interpreted with caution and large prospective studies are still required to further investigate these findings.

## 1. Introduction

Lung cancer is currently the most common human malignancies and the leading cause of cancer-related mortality in the world. Non-small cell lung cancer (NSCLC) accounts for ~85% of all lung cancer cases, with 30% being squamous cell carcinoma (SCC) and the remaining (~70%) being collectively classified as non-SCC [1]. Despite efforts for early detection, approximately two-thirds of NSCLC are diagnosed at advanced stages with limited surgery options. Recent advances in target therapy in NSCLC have added more choices for non-SCC, but chemotherapy still remains the therapeutic mainstay for SCC and some non-SCC. Platinum combined with the third-generation cytotoxic drugs including gemcitabine and pemetrexed is the standard first-line treatment for advanced NSCLC [2]. However, the chemotherapy response and clinical prognosis for NSCLC patients vary remarkably among individuals. Some patients show significant tumor regression while others may develop intrinsic or acquired resistance to chemical drugs, which highlights the need to tailor the treatment for individuals [3]. Pharmacogenomics has been perceived as a useful tool to predict treatment response as well as clinical prognosis in cancer patients, and numerous studies have suggested the functions of single nucleotide polymorphisms (SNPs) in affecting drug sensitivity by modifying relevant genes [4]. Consequently, it is necessary to identify functional gene polymorphisms as new biomarkers for accurately predicting clinical outcomes of chemotherapy in NSCLC, which could also reduce the side effects and provide the most cost-effective approach for individuals [3, 4].

The cytotoxic mechanism of platinum is that the agents bind to DNA and form platinum-DNA adducts, which consequently block DNA replication and transcription, resulting in cellular apoptosis and growth inhibition. The damaged DNA system could be repaired by many biological processes, leading to a resistance to the platinum. The nucleotide excision repair (NER) pathway plays dominant roles in the DNA repair process, which is responsible for the recognition of DNA damage and removal of the damaged nucleotides [5]. Excision repair cross-complementation group 1 (ERCC1) is a key rate-limiting enzyme in the NER pathway. It has been documented that high expression of ERCC1 could block the platinum efficacy and cause drug resistance [6]. Therefore, ERCC1 has become one of the most promising biomarkers for efficacy of platinum-based chemotherapy. Two most common SNPs of ERCC1 gene are C118T (rs11615) with a C → T substitution at exon 4 and C8092A (rs3212986) with a C→A change in the 3′-untranslated region, both of which have been speculated to regulate the ERCC1 mRNA expression and manage the NER pathway, consequently affecting the efficacy of platinum-based chemotherapy [7].

To date, lots of studies regarding gene variants that may alter response and tolerability to chemotherapy drugs have been carried out, including both retrospective and perspective studies. The ultimate purpose of a pharmacogenomics analysis is to evaluate the potential value of individual's gene variants to predict the drug efficacy, thus allowing for optimization and personalization of the clinical decision making. However, such an approach seems still a distant goal. The previous results on the relationships between ERCC1 polymorphisms and clinical outcomes of platinum-based chemotherapy in NSCLC are inconclusive and even conflicting to each other, probably due to the complex gene interaction, environmental effects, different detection methods, sample sizes, or study designs. Therefore, we performed a systematic review and meta-analysis of published studies to comprehensively evaluate the predictive values of two promising ERCC1 SNPs for clinical outcomes of platinum-based chemotherapy in NSCLC, with an expectation to provide useful evidence and suggestions for clinical practice and future investigation.

## 2. Materials and Methods

### 2.1. Literature Search

We searched for original studies investigating the associations between ERCC1 SNPs and platinum-based chemotherapy in NSCLC by using MEDLINE and EMBASE databases. The following terms were combined variously for search: “ERCC1,” “platinum or cisplatin or carboplatin,” “polymorphism or variant or mutant,” and “lung cancer.” The literature search was last updated on April 2015. References from retrieved articles and previous meta-analysis were further screened manually for additional qualified studies.

### 2.2. Inclusion Criteria

The inclusion criteria were as follows: (1) patients should be pathologically confirmed to have NSCLC; (2) studies should assess the relationships between ERCC1 polymorphisms and clinical outcomes of platinum-based chemotherapy; (3) polymorphisms should be genotyped: ERCC1 C118T (rs11615), C8092A (rs3212986); and (4) clinical outcomes including the objective response rate (ORR), overall survival (OS), and progression-free survival (PFS) should be reported. The most recent and comprehensive data were included if there were duplications.

### 2.3. Quality Assessment

The quality of each study was assessed independently by two reviewers (Han and Liu) using the Newcastle-Ottawa Scale (NOS), and disputations were settled by a third reviewer (Sun). The NOS consists of 3 parameters for the quality of case-control/cohort study: selection, comparability, and exposure/outcome. Studies with NOS scores >6 were considered with high quality [8].

### 2.4. Data Extraction

Data extraction was performed independently by two investigators (Han and Liu) and disagreements were adjudicated by a third reviewer (Sun). For each study, general characteristics such as the first author, publication year, country and ethnicity of patients, sample size, tumor stages, follow-up time, chemotherapy drugs and treatment line, SNP allele frequency, and genotyping method were collected. Results data included odds ratio (OR) for the ORR, hazard ratios (HR), and 95% confidence interval (CI) for the OS and PFS. If HR and 95% CI were not available directly, the estimated value was derived indirectly using Tierney's methods [9].

### 2.5. Statistic Analysis

The ORR was used to measure the efficacy of chemotherapy regimens. Patients were divided into the responder group including the complete responders (CR) or partial responders (PR) and the nonresponders group including the stable disease (SD) or progressive disease (PD) according to the Response Evaluation Criteria in Solid Tumors criteria [10]. The pooled OR and 95% CI were calculated by (CR + PR)/(SD + PD). The OS and PFS on behalf of prognosis of NSCLC patients were evaluated by calculating pooled Cox proportional HR and 95% CI as relevant effect measures. The associations between SNPs and ORR/OS/PFS were examined by both the dominant (heterozygote or homozygote variant versus wild type) and codominant models (heterozygote variant versus wild type, homozygote variant versus wild type). Heterogeneity among studies was tested by the Chi-square-based *Q* test and *I* ^2^ statistics. Values of *P* > 0.10 for the *Q* test or *I* ^2^ < 50% were considered as lack of heterogeneity and a fixed-effect model was used; otherwise, a random-effect model was used. Subgroup analysis by ethnicity was used to detect and reduce potential source of heterogeneity among studies. The publication bias was investigated by the inverted funnel plot, Begg's test, and Egger's test. Values of *P* < 0.05 were indicative of statistically significant publication bias. All *p* values were two sided. All analyses were performed using the Review Manager version 5.3 (Oxford, England) and the Stata version 12.0 (Stata Corporation, College Station, TX).

## 3. Results

### 3.1. Study Identification

**Figure 1:**
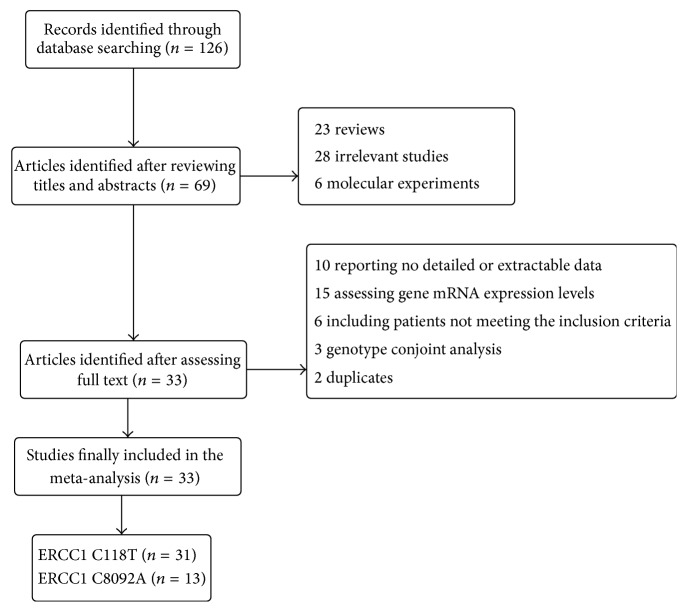
Flow chart for the study selection process.

As shown in the flow chart for the study selection process (Figure 1), 126 publications were initially retrieved from the PUBMED and EMBASE databases. After screening by checking title and abstract, 57 articles being reviews, meta-analyses, or molecular experiments and other irrelevant studies were excluded. Then, we checked full text for the remaining 69 studies, and subsequently 36 studies were excluded for the following reasons: evaluating the association between gene expression levels and clinical outcome, including patients with SCLC or other tumors, including patients treated with chemotherapy regimens without platinum, conducting the genotype conjoint analysis, and reporting no detailed or extractable survival data. Finally, a total of 33 studies including 5373 patients were identified to be eligible for this meta-analysis. All of our included studies had a high quality with NOS scores >6. The baseline characteristics of the included studies were shown in Table 1 and all the results were summarized in Table 2.

### 3.2. ERCC1 C118T

#### 3.2.1. Objective Response Rate

**Figure 2:**
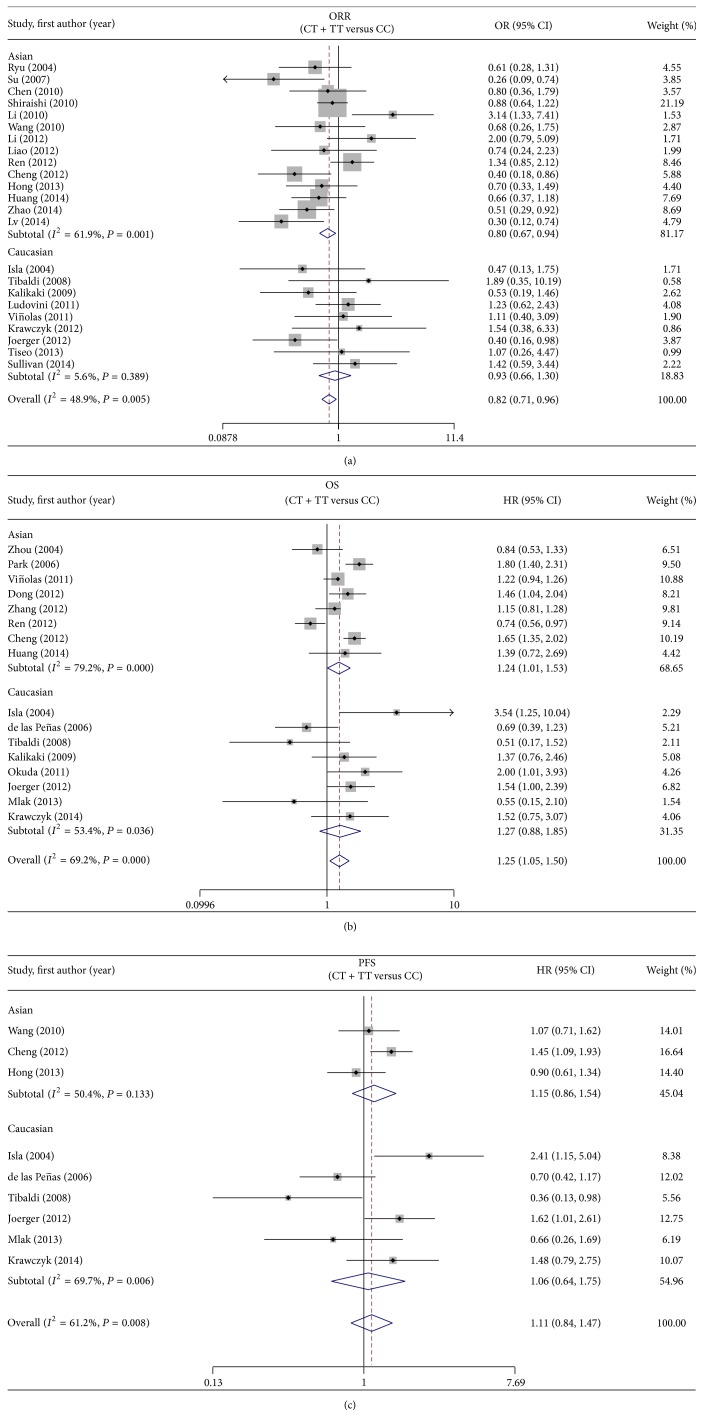
Forest plot of (a) ORR, (b) OS, and (c) PFS in NSCLC patients treated with platinum-based chemotherapy in relation to ERCC1 C118T polymorphism (CT + TT versus CC).

Twenty-three studies including 3272 patients were eligible for this analysis [11–33]. Overall, the T allele showed a significant association with a poorer ORR (CT + TT versus CC: OR = 0.82, 95% CI = 0.71–0.96, *I* ^2^ = 48.9%, and *P*_heterogeneity_ = 0.005) (Figure 2(a)). For the ethnicity-specific subgroup analysis, the T allele was found to be correlated with a decreased ORR in Asian patients, but not in Caucasian patients (CT + TT versus CC: for Asians, OR = 0.80, 95% CI = 0.67–0.94, *I* ^2^ = 61.9%, and *P*_heterogeneity_ = 0.01; for Caucasians: OR = 0.93, 95% CI = 0.66–1.30, *I* ^2^ = 5.6%, and *P*_heterogeneity_ = 0.389) (Figure 2(a)). Similar results were found by the codominant model (CT versus CC: for total, OR = 0.73, 95% CI = 0.61–0.89, *I* ^2^ = 9%, and *P*_heterogeneity_ = 0.35; for Asians, OR = 0.69, 95% CI = 0.55–0.85, *I* ^2^ = 0%, and *P*_heterogeneity_ = 0.44; for Caucasians, OR = 0.93, 95% CI = 0.62–1.38, *I* ^2^ = 16%, and *P*_heterogeneity_ = 0.31) (TT versus CC: for total, OR = 0.63, 95% CI = 0.47–0.85, *I* ^2^ = 19%, and *P*_heterogeneity_ = 0.24; for Asians, OR = 0.64, 95% CI = 0.44–0.93, *I* ^2^ = 20%, and *P*_heterogeneity_ = 0.27; for Caucasians, OR = 0.63, 95% CI = 0.39–1.01, *I* ^2^ = 30%, and *P*_heterogeneity_ = 0.20) (Table 2).

#### 3.2.2. Overall Survival

Seventeen studies including 2926 patients were available for this evaluation [11, 14, 15, 21, 22, 25, 27, 32–41]. Generally, patients with CT or TT genotype had a poorer OS when compared to those with CC genotype (CT + TT versus CC: HR = 1.25, 95% CI = 1.05–1.50). However, significant heterogeneity (*I* ^2^ = 69.2%, *P*_heterogeneity_ = 0.000) was found in this analysis and the random effect model was applied (Figure 2(b)). In the ethnic subgroup analysis, CT or TT genotype was found to be associated with an unfavorable OS in Asians but not in Caucasians (CT + TT versus CC: for Asians, HR = 1.24, 95% CI = 1.01–1.53, *I* ^2^ = 79.2%, and *P*_heterogeneity_ = 0.000; for Caucasians, HR = 1.27, 95% CI = 0.88–1.85, *I* ^2^ = 53.4%, and *P*_heterogeneity_ = 0.036) (Figure 2(b)). Obvious heterogeneity still existed and sensitivity analysis indicated no study to be deleted. In the codominant model analysis with only Asians, the homozygote showed a more significant trend to be associated with survival than the heterozygote (CT versus CC: HR = 1.09, 95% CI = 0.86–1.38, *I* ^2^ = 50%, and *P*_heterogeneity_ = 0.09) (TT versus CC: HR = 1.49, 95% CI = 1.18–1.88, *I* ^2^ = 48%, and *P*_heterogeneity_ = 0.11) (Table 2).

#### 3.2.3. Progression-Free Survival

Nine studies with 943 patients were included in this analysis [11, 14, 19, 25, 27, 29, 36, 40, 41]. There was no evidence to support an association between the ERCC1 C118T polymorphism and PFS in the dominant model (CT + TT versus CC: HR = 1.11, 95% CI = 0.84–1.47, *I* ^2^ = 61.2%, and *P*_heterogeneity_ = 0.008) (Figure 2(c)). The ethnic subgroup analysis also demonstrated no relationship between the ERCC1 C118T polymorphism and PFS in Asians or Caucasians (CT + TT versus CC: for Asians, HR = 1.15, 95% CI = 0.86–1.54, *I* ^2^ = 50.4%, and *P*_heterogeneity_ = 0.133; for Caucasians, HR = 1.06; 95% CI = 0.64–1.75, *I* ^2^ = 69.7%, and *P*_heterogeneity_ = 0.006) (Figure 2(c)). Only one study conducted this evaluation in the codominant model (CT versus CC: HR = 1.47, 95% CI = 0.91–2.36; TT versus CC: HR = 1.86, 95% CI = 1.13–3.07), and the homozygote showed a more obvious trend to be associated with PFS than the heterozygote (Table 2).

### 3.3. ERCC1 C8092A

#### 3.3.1. Objective Response Rate

**Figure 3:**
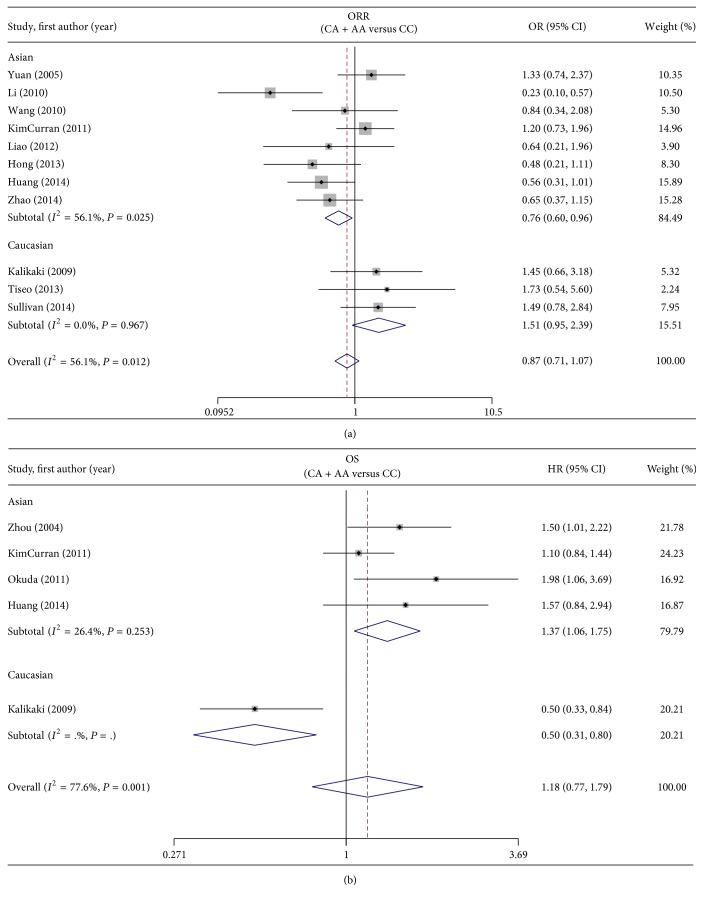
Forest plot of (a) ORR and (b) OS in NSCLC patients treated with platinum-based chemotherapy in relation to ERCC2 C8092A polymorphism (CA + AA versus CC).

The data from eleven studies with 1607 patients were included in this evaluation [15, 16, 19, 24, 28, 29, 31–33, 42, 43]. In total, no statistically significant association was found between ERCC1 C8092A and ORR (CA + AA versus CC: OR = 0.87, 95% CI = 0.71–1.07, *I* ^2^ = 56.1%, and *P*_heterogeneity_ = 0.012) (Figure 3(a)). Stratified analysis by ethnicity showed an obvious association in Asians, but not in Caucasians (CA + AA versus CC: for Asians, OR = 0.76, 95% CI = 0.60–0.96, *I* ^2^ = 56.1%, and *P*_heterogeneity_ = 0.025; for Caucasians, OR = 1.51, 95% CI = 0.95–2.39, *I* ^2^ = 0%, and *P*_heterogeneity_ = 0.967) (Figure 3(a)). Positive results were also found by the codominant model in Asian patients (CA versus CC: OR = 0.71, 95% CI = 0.53–0.96, *I* ^2^ = 57%, and *P*_heterogeneity_ = 0.04) (AA versus CC: OR = 0.42, 95% CI = 0.26–0.70, *I* ^2^ = 0%, and *P*_heterogeneity_ = 0.57) (Table 2).

#### 3.3.2. Overall Survival and Progression-Free Survival

Seven studies consisting of 1078 patients were selected for this analysis [15, 24, 32–34, 37, 42]. Overall, no correlation was observed between ERCC1 C8092A and survival (CA + AA versus CC: HR = 1.18, 95% CI = 0.77–1.79), and high level of heterogeneity was also observed in this analysis (*I* ^2^ = 77.6%, *P*_heterogeneity_ = 0.001) (Figure 3(b)). After excluding the only one study for Caucasians, A allele was observed to be associated with a worse survival in the remaining four Asian studies (HR = 1.37, 95% CI = 1.06–1.75, *I* ^2^ = 26.4%, and *P*_heterogeneity_ = 0.253). Significant association was also found by the codominant model in Asian patients (CA versus CC: HR = 1.38, 95% CI = 1.03–1.86, *I* ^2^ = 0%, and *P*_heterogeneity_ = 0.41) (AA versus CC: HR = 2.03, 95% CI = 1.19–3.47, *I* ^2^ = 54%, and *P*_heterogeneity_ = 0.09) (Table 2). Only one Chinese study with 90 patients examined the relationship between ERCC1 C8092A and PFS, and no association was observed (HR = 1.08, 95% CI = 0.72–1.63) (Table 2).

### 3.4. Publication Bias

The publication bias was performed in the evaluation of the relationship between ERCC1 C118T and ORR by the dominant model, since this comparison included the most studies (*n* = 23). The funnel plot revealed no obvious publication bias, with a symmetrical distribution of study results around the pooled measurement of effect. No evidence of publication bias was detected according to Begg's test (*P* = 1.000) and Egger's test (*P* = 0.685).

## 4. Discussion

The optimal treatment for cancer patients has been evolving for decades. Lots of studies have demonstrated the significant association between gene characteristics and chemotherapy efficacy in individuals. With respect to the gene characteristics, gene polymorphisms are considered more accessible for detection and valuable for evaluation than gene mRNA levels. Single nucleotide polymorphisms (SNPs) have been widely suggested to be capable of affecting the drug sensitivity by modifying functions of relevant genes, thus being predictive biomarkers for treatment efficacy in cancer patients. ERCC1 has been well known to be a promising screening tool in the application of platinum-based chemotherapy in NSCLC. Numerous studies have been carried out to address this question and quite a few meta-analyses evaluating this question have been published. However, to date, no definitive conclusion has been reached yet. The latest meta-analysis was conducted by Yang and Xian who did the literature search to July 31, 2013 [44]. We updated this meta-analysis to evaluate the issue more comprehensively with an expectation to get some statistical advancement. We hope our efforts to select gene biomarkers could make a positive contribution to the progress of optimization and personalization of chemotherapy in NSCLC.

Yang and Xian demonstrated merely a trend of ERCC1 C118T in predicting ORR with no statistical significance (CT + TT versus CC: OR = 0.94, 95% CI = 0.72–1.23), and stratified analysis by ethnicity also failed to show any valuable results. Besides, they got an opposite trend when analyzing the relevance of C8092A with ORR by the dominant model, probably due to the heterogeneity resulting from ethnicity (CA + AA versus CC: for total: OR = 1.05, 95% CI = 0.83–1.32; for Asians: OR = 0.97, 95% CI = 0.75–1.25; for Caucasians: OR = 1.55, 95% CI = 0.87–2.74). The assessment by the codominant model did not reveal any significant results either. Notably, our updated analysis with more data exactly showed a significant relevance of T allele and A allele with a worse ORR compared with C allele in the Asian subgroup, by both the dominant and codominant models. Our statistically significant results highlight a good prospect of these two ERCC1 SNPs as predictive biomarkers for the response of platinum-based chemotherapy in NSCLC.

Furthermore, when assessing the predictive values of ERCC1 C118T/C8092A for prognosis of NSCLC patients receiving platinum-based chemotherapy, we also obtained some statistical advancements. Yang and Xian observed an association between C118T and OS in totals and in Asians (CT + TT versus CC, for total: HR = 1.26, 95% CI = 1.02–1.55, *I* ^2^ = 67%, and *P*_heterogeneity_ < 0.001; for Asians: HR = 1.35, 95% CI = 1.04–1.75, *I* ^2^ = 73.3%, and *P*_heterogeneity_ = 0.001; for Caucasians: HR = 1.12, 95% CI = 0.74–1.68, *I* ^2^ = 63%, and *P*_heterogeneity_ = 0.019), while no apparent correlation between C8092A and OS in both ethnic groups was observed (CA + AA versus CC, for total: HR = 1.26, 95% CI = 0.81–1.95, *I* ^2^ = 87%, and *P*_heterogeneity_ < 0.001; for Asians: HR = 1.50, 95% CI = 0.89–2.55, *I* ^2^ = 87.4%, and *P*_heterogeneity_ < 0.001; for Caucasians: HR = 0.87, 95% CI = 0.30–2.56, *I* ^2^ = 91.9%, and *P*_heterogeneity_ < 0.001). The great heterogeneity in Yang's analysis to some extent impacted the credibility of their results. Importantly, in our analysis for C8092A, A allele exhibited a significant association with a poorer OS in the Asian subgroup analysis with smaller heterogeneity (CA + AA versus CC, for Asians: HR = 1.37, 95% CI = 1.06–1.75, *I* ^2^ = 26.4%, and *P*_heterogeneity_ = 0.253), and consistent results were observed by the codominant model, which indicated a possibility that C8092A may serve as a predictor for clinical prognosis of platinum-based chemotherapy in NSCLC. Nevertheless, in the C118T analysis, we revealed similar results with Yang's analysis and significant heterogeneity still existed by the dominant model. Notably, in our evaluation by the codominant model which only included Asian patients, the homozygote showed obvious association with an unfavorable OS while the heterozygote only showed a slight trend without statistical significance. This phenomenon presents a possibility that the number of T alleles may be inversely proportional to the clinical outcome of platinum-based chemotherapy.

Fewer studies reported data of PFS, and most of them performed analysis by the dominant model. Combining the extracted data, we could not identify a predictive role of ERCC1 C118T/C8092A for PFS of platinum-based chemotherapy in NSCLC. However, we also observed a slight trend that the T allele may correlate with a poorer PFS, as the HRs in various comparisons of mutant genotype with wild genotype all showed greater than 1 (CT + TT versus CC, HR = 1.11; CT versus CC, HR = 1.47; and TT versus CC, HR = 1.86). Only one study reported the relevance of C8092A with PFS, which also revealed no obvious association. In consideration of the positive results obtained in the evaluation for ORR and OS, there still exists the possibility that minor variants in ERCC1 SNPs can as well predict PFS. More studies in the future are expected to confirm this possibility.

In particular consideration of the influence of race on chemosensitivity, we conducted the stratified analysis by ethnicity. Notably, the results revealed a significant distinction between the Asian and the Caucasian subgroups, with ERCC1 SNPs predicting both ORR and OS prominently in Asian patients but not in Caucasian patients. The significant ethnic discrepancy in the predictive roles of ERCC1 SNPs may be due to the gene-gene and gene-environment interactions, different genetic background, and lifestyle, which is not yet clearly defined.

Our work has several strengths. First, we comprehensively evaluated gene effects on the standard first-line treatment in NSCLC, platinum-based chemotherapy, and the indicators covered both treatment efficacy and clinical prognosis (ORR, OS, and PFS); second, we made a statistical progress when evaluating the predictive values of ERCC1 polymorphisms for platinum-based chemotherapy in NSCLC; third, all of our included studies were of high quality and no evident publication bias was observed. All these points significantly increased the statistical power of our analysis.

Despite the strengths of our work, some limitations should be taken into consideration. Above all, an obvious heterogeneity was observed in the analysis of the ERCC1 C118T for OS and PFS and still existed after stratified analysis by ethnicity. It is speculated that the heterogeneity mainly derived from heterogeneous samples and various clinical trial designs: firstly, the baseline characteristics of populations in different trials were impossible to coincide with each other, such as age, sex, histology type, and tumor stage; secondly, clinical trial designs varied in many aspects, such as the coadministration of other drugs, drug administration mode, treatment cycle, treatment line with or without surgery or radiotherapy, and genotyping method. Most of the included studies employed the platinum-based chemotherapy as a first-line treatment; only a few studies did not specialize the treatment line. And the concurrent drugs combined in the platinum-based regimen also to some extent affected the clinical outcome, as the efficacy of gemcitabin and pemetrexed also varied among individuals and could be influenced by certain gene polymorphisms as well, such as ribonucleotide reductase M1(RRM1) and thymidylate synthase (TS). All of these potential discrepancies interfere with the standardization of data acquisition; however, we were unable to conduct a further stratified analysis since most studies did not provide detailed information about these factors. In addition, there inevitably existed some inaccuracies in our data extraction and analysis: firstly, some researches in the literature did not report concrete time-to-event data so that we had to obtain calculable data indirectly by reading Kaplan–Meier curves or conducting data transformation; secondly, most studies reported unadjusted estimates and only a few studies are supplied with adjusted estimates; moreover, those estimates were not always adjusted for the same potential confounders.

We still have a long way to go to ultimately turn to ERCC1 polymorphisms for our clinical decision making, since various factors contribute to the individual variation in drug response. Polymorphism of genes encoding proteins involved in the transport, metabolism, and action of drugs influences the clinical outcome of chemotherapies prominently, and other factors such as age, sex, physical condition, hepatic and renal function, tolerance to toxicity, and alcohol and tobacco use also affect the drug efficacy. All the factors should be taken into account comprehensively when making clinical decisions. Although it is a distant goal, our work has made an important step along this path, indicating a necessity to conduct future prospective studies with large sample sizes and better study designs to validate these conclusions and prove the feasibility of the customization approach to our clinical decision making.

## 5. Conclusion

In conclusion, the prospect of optimal chemotherapy in NSCLC based on validated biomarkers was further confirmed with the latest data. We provided statistical evidence that both ERCC1 C118T and C8092A could be useful predictive biomarkers for platinum-based chemotherapy in Asian NSCLC patients. However, considering the limitations, heterogeneity and biases existed within our analysis, our conclusions should be interpreted with caution, and large prospective studies are still required to further validate these findings.

## Figures and Tables

**Table 1:** Baseline characteristics of included studies.

First author	Year	Ethnicity (country)	Case number	Median age	Disease stage	Follow-up time	Treatment line	Chemotherapy	Outcomes	Genotyping method	SNPs (allele frequency)
Zhou [34]	2004	Caucasian (USA)	128	60 (32–78)	III-IV	65.3 (4.9–121.5)	1st/2nd	Platinum-based	OS	PCR-RFLP	rs11615 (T: 0.60), rs3212986 (A: 0.32)
Isla [11]	2004	Caucasian (Spain)	62	62 (35–78)	IIIB-IV	13.5 (8–19)	1st	Cisplatin/docetaxel	ORR, OS, PFS	TaqMan PCR	rs11615 (T: 0.55)
Ryu [12]	2004	Asian (Korea)	109	60 (32–78)	IIIB-IV	NR	1st	Cisplatin based	ORR	SNaPShot assay	rs11615 (T: 0.28)
Yuan [43]	2005	Asian (China)	200	56 (30–74)	IIIB-IV	NR	1st	Cis or carboplatin	ORR	PCR-RFLP	rs3212986 (A: 0.26)
de las Peñas [36]	2006	Caucasian (Spain)	135	62 (31–81)	IIIB-IV	9.7 (0.4–30.7)	1st	Cisplatin/gemcitabine	OS, PFS	TaqMan PCR	rs11615 (T: 0.57)
Park [35]	2006	Asian (Korea)	245	67 (28–92)	III-IV	NR	1st	Cisplatin based	OS	SNaPShot assay	rs11615 (T: 0.24)
Su [13]	2007	Asian (China)	76	58 (28–80)	III-IV	NR	1st	Platinum-based	ORR	TaqMan PCR	rs11615 (T: 0.25)
Tibaldi [14]	2008	Caucasian (Italy)	65	65 (44–77)	IIIB-IV	NR	1st	Cisplatin/gemcitabine	ORR, OS, PFS	TaqMan PCR	rs11615 (T: 0.58)
Kalikaki [15]	2009	Caucasian (Greece)	119	61 (39–85)	III-IV	28.5 (0.4–60)	1st/2nd	Platinum-based	ORR, OS	PCR-RFLP	rs11615 (T: 0.5), rs3212986
Li [16]	2010	Asian (China)	115	59.6	IIIB-IV	NR	1st	Cis or carboplatin	ORR	3D polyacrylamide gel-based DNA microarray	rs11615 (T: 0.19), rs3212986 (A: 0.35)
Shiraishi [17]	2010	Asian (Japan)	640	58 (22–78)	IIIB-IV	NR	1st	Platinum-based	ORR	Pyrosequencing or TaqMan PCR	rs11615 (T: 0.27)
Chen [18]	2010	Asian (China)	95	58 (35–77)	IIIB-IV	NR	NS	Cisplatin based	ORR	Ligase detection reactions	rs11615 (T: 0.26)
Wang [19]	2010	Asian (China)	90	55 (33–73)	IIIB-IV	9 (3–15)	1st	Cisplatin based	ORR, PFS	PCR-based sequencing	rs11615 (T: 0.24), rs3212986 (A: 0.35)
KimCurran [42]	2011	Asian (China)	300	60 (33–78)	IIIB-IV	NR	1st	Cis or carboplatin	ORR, OS	TaqMan PCR	rs3212986 (A: 0.35)
Okuda [37]	2011	Asian (Japan)	90	60	I–IV	60	NS	Cis or carboplatin	OS	PCR-RFLP	rs11615 (T: 0.3), rs3212986 (A: 0.19)
Ludovini [20]	2011	Caucasian (Italy)	192	63 (25–81)	IIIB-IV	24.6 (14.5–40.8)	1st	Cisplatin/gemcitabine	ORR	TaqMan PCR	rs11615 (T: 0.44)
Viñolas [21]	2011	Caucasian (Spain)	94	61 (37–77)	IIIB-IV	NR	1st	Cisplatin/vinorelbine	ORR	TaqMan PCR	rs11615 (T: 0.59)
Ren [22]	2012	Asian (China)	340	60 (30–78)	IIIB-IV	18 (8–66)	1st	Cis or carboplatin	ORR, OS	TaqMan PCR	rs11615
Li [23]	2012	Asian (China)	89	59 (21–84)	III-IV	NR	NS	Cisplatin based	ORR	TaqMan PCR	rs11615
Liao [24]	2012	Asian (China)	62	57 (36–78)	IIIB-IV	22 (4–65)	1st	Platinum-based	ORR, OS	PCR-RFLP	rs11615 (T: 0.24), rs3212986 (A: 0.40)
Joerger [25]	2012	Caucasian (Holland)	137	59.7 (37–79)	IIIB-IV	40.00	1st	Platinum/gemcitabine	ORR, OS, PFS	TaqMan PCR	rs11615 (T: 0.60)
Dong [38]	2012	Asian (China)	181	NR	III-IV	18.80	1st	Platinum-based	OS	Illumina high-throughput genotyping	rs11615 (T: 0.23)
Krawczyk [26]	2012	Caucasian (Poland)	43	63	IIIB-IV	NR	1st	Cis or carboplatin	ORR	PCR-RFLP	rs11615 (T: 0.45)
Cheng [27]	2012	Asian (China)	142	62 (43–81)	IIIB-IV	NR	1st	Cisplatin based	ORR, OS, PFS	AllGlo Probe-based RT-PCR	rs11615 (T: 0.23)
Zhang [39]	2012	Asian (China)	632	62.6	I–IV	31.60	1st	Cisplatin/gemcitabine	OS	TaqMan PCR	rs11615 (T: 0.59)
Tiseo [28]	2013	Caucasian (Italy)	49	63 (29–79)	IIIB-IV	NR	1st	Cisplatin based	ORR	TaqMan PCR	rs11615, rs3212986
Hong [29]	2013	Asian (China)	135	56 (25–72)	IIIB-IV	NR	1st	Platinum/gemcitabine	ORR, PFS	TaqMan PCR	rs11615 (T: 0.3), rs3212986 (A: 0.34)
Mlak [40]	2013	Caucasian (Poland)	62	61 (38–76)	III-IV	NR	1st	Platinum/gemcitabine	OS, PFS	PCR-RFLP	rs11615 (T: 0.66)
Lv [30]	2014	Asian (China)	91	59 (34–80)	IIIB-IV	8 (0.5–13.5)	NS	Cisplatin based	ORR	TaqMan PCR	rs11615 (T: 0.29)
Sullivan [31]	2014	Caucasian (Spain)	161	63.7 (36–85)	III-IV	NR	NS	Cis or carboplatin	ORR	TaqMan PCR	rs11615 (T: 0.66), rs3212986 (A: 0.22)
Zhao [32]	2014	Asian (China)	192	60.8 (26–79)	III-IV	48 (36–60)	NS	Platinum-based	ORR, OS	MassARRAY Analyzer 4 system	rs11615 (T: 0.36), rs3212986 (A: 0.32)
Krawczyk [41]	2014	Caucasian (Poland)	115	61	II–IV	50 (26–74)	1st	Platinum/pemetrexed	OS, PFS	High Resolution Melt (HRM)	rs11615 (T: 0.61)
Huang [33]	2014	Asian (China)	187	63.8	III-IV	39 (27–51)	1st	Platinum-based	ORR, OS	MassARRAY Analyzer system	rs11615 (T: 0.33), rs3212986 (A: 0.28)

NR: not reported, NS: not specialized.

**Table 2:** Summary data on associations between ERCC1 polymorphisms and ORR, OS, and PFS of platinum-based chemotherapy in NSCLC.

	Study (cases)	Total	Study (cases)	Asian	Study (cases)	Caucasian
ERCC1 C118T						
ORR						
CT + TT versus CC	23 (3272)	0.82 [0.71, 0.96]	14 (2361)	0.80 [0.67, 0.94]	9 (911)	0.93 [0.66, 1.30]
		*I* ^2^ = 48.9%, *P*_heterogeneity_ = 0.005		*I* ^2^ = 61.9%, *P*_heterogeneity_ = 0.001		*I* ^2^ = 5.6%, *P*_heterogeneity_ = 0.389
CT versus CC	16 (2023)	0.73 [0.61, 0.89]	9 (1499)	0.69 [0.55, 0.85]	7 (524)	0.93 [0.62, 1.38]
		*I* ^2^ = 9%, *P*_heterogeneity_ = 0.35		*I* ^2^ = 0%, *P*_heterogeneity_ = 0.44		*I* ^2^ = 16%, *P*_heterogeneity_ = 0.31
TT versus CC	16 (1314)	0.63 [0.47, 0.85]	9 (985)	0.64 [0.44, 0.93]	7 (329)	0.63 [0.39, 1.01]
		*I* ^2^ = 19%, *P*_heterogeneity_ = 0.24		*I* ^2^ = 20%, *P*_heterogeneity_ = 0.27		*I* ^2^ = 30%, *P*_heterogeneity_ = 0.20
OS						
CT + TT versus CC	16 (2734)	1.25 [1.05, 1.50]	8 (1949)	1.24 [1.01, 1.53]	8 (785)	1.27 [0.88, 1.85]
		*I* ^2^ = 69.2%, *P*_heterogeneity_ = 0.000		*I* ^2^ = 79.2%, *P*_heterogeneity_ = 0.000		*I* ^2^ = 53.4%, *P*_heterogeneity_ = 0.036
CT versus CC	5 (1276)	1.09 [0.86, 1.38]	5 (1276)	1.09 [0.86, 1.38]	0
		*I* ^2^ = 50%, *P*_heterogeneity_ = 0.09		*I* ^2^ = 50%, *P*_heterogeneity_ = 0.09	
TT versus CC	5 (1276)	1.49 [1.18, 1.88]	5 (1276)	1.49 [1.18, 1.88]	0
		*I* ^2^ = 48%, *P*_heterogeneity_ = 0.11		*I* ^2^ = 48%, *P*_heterogeneity_ = 0.11	
PFS						
CT + TT versus CC	9 (943)	1.11 [0.84, 1.47]	3 (367)	1.15 [0.86, 1.54]	6 (576)	1.06 [0.64, 1.75]
		*I* ^2^ = 61.2%, *P*_heterogeneity_ = 0.008		*I* ^2^ = 50.4%, *P*_heterogeneity_ = 0.133		*I* ^2^ = 69.7%, *P*_heterogeneity_ = 0.006
CT versus CC	1 (137)	1.47 [0.91, 2.36]	1 (137)	1.47 [0.91, 2.36]	0	
TT versus CC	1 (137)	1.86 [1.13, 3.07]	1 (137)	1.86 [1.13, 3.07]	0	
ERCC1C8092A						
ORR						
CA + AA versus CC	11 (1607)	0.87 [0.71, 1.07]	8 (1279)	0.76 [0.60, 0.96]	3 (328)	1.51 [0.95, 2.39]
		*I* ^2^ = 56.1%, *P*_heterogeneity_ = 0.012		*I* ^2^ = 56.1%, *P*_heterogeneity_ = 0.025		*I* ^2^ = 0%, *P*_heterogeneity_ = 0.967
CA versus CC	6 (793)	0.71 [0.53, 0.96]	6 (793)	0.71 [0.53, 0.96]	0
		*I* ^2^ = 57%, *P*_heterogeneity_ = 0.04		*I* ^2^ = 57%, *P*_heterogeneity_ = 0.04	
AA versus CC	6 (529)	0.42 [0.26, 0.70]	6 (529)	0.42 [0.26, 0.70]	0
		*I* ^2^ = 0%, *P*_heterogeneity_ = 0.57		*I* ^2^ = 0%, *P*_heterogeneity_ = 0.57	
OS						
CA + AA versus CC	5 (824)	1.18 [0.77, 1.79]	4 (705)	1.37 [1.06, 1.75]	1 (119)	0.50 [0.33, 0.84]
		*I* ^2^ = 77.6%, *P*_heterogeneity_ = 0.001		*I* ^2^ = 26.4%, *P*_heterogeneity_ = 0.253		
CA versus CC	4 (569)	1.38 [1.03, 1.86]	4 (569)	1.38 [1.03, 1.86]	0
		*I* ^2^ = 0%, *P*_heterogeneity_ = 0.41		*I* ^2^ = 0%, *P*_heterogeneity_ = 0.41	
AA versus CC	4 (569)	2.03 [1.19, 3.47]	4 (569)	2.03 [1.19, 3.47]	0
		*I* ^2^ = 54%, *P*_heterogeneity_ = 0.09		*I* ^2^ = 54%, *P*_heterogeneity_ = 0.09	
PFS						
CA + AA versus CC	1 (90)	1.08 [0.72, 1.63]	1 (90)	1.08 [0.72, 1.63]		
